# Characteristics of patients who developed transient anti-adalimumab antibodies

**DOI:** 10.1186/s12348-025-00520-7

**Published:** 2025-09-24

**Authors:** Osama Elaraby, Dalia El Feky, Cigdem Yasar, Woong-Sun Yoo, Anadi Khatri, Dalia Abd Elmegid, Jia-Horung Hung, Battuya Ganbold, Zheng Xian Thng, Negin Yavari, Aim-On Saengsirinavin, Ngoc Trong Tuong Than, Frances Andrea Anover, Abdelrahman M. Hamouda, S. Saeed Mohammadi, Irmak Karaca, Amir Akhavanrezayat, Anh Tram Ngoc Tran, Jingli Guo, Yue Bai, Quan Dong Nguyen, Christopher Or

**Affiliations:** 1 Department of Ophthalmology, Byers Eye Institute, Stanford University, 2452 Watson Court, Suite 200, Palo Alto, CA 94303 USA; 2 Department of Public Health and Community Medicine, Tanta University, Tanta, Egypt; 3 Bolor Melmii Eye Hospital, Ulaanbaatar, Mongolia; 4 Department of Neurological Surgery, Mayo Clinic, Rochester, MN USA

**Keywords:** Adalimumab, Anti-adalimumab antibodies, Uveitis, Rheumatoid arthritis, Crohn’s disease

## Abstract

**Background:**

Adalimumab is a monoclonal antibody approved for the treatment of autoimmune diseases and non-infectious uveitis (NIU). It targets tumor necrosis factor alpha, a key mediator in inflammation. However, the development of anti-adalimumab antibodies (AAA) can reduce therapeutic efficacy and prompt treatment modifications. This study aimed to describe the clinical characteristics of patients with transient AAA and compare them to patients with persistent AAA, testing whether serum antibody and drug levels differ between groups.

**Main body:**

We conducted a retrospective cohort study using the Stanford Research Repository (STARR) to identify patients treated with adalimumab for autoimmune conditions between June 2006 and May 2024 who developed AAA. Patients whose AAA became undetectable on follow-up testing were compared to an age-, sex-, and disease-matched cohort with persistent AAA. Demographics, diagnoses, treatment details, serum adalimumab and AAA levels, and concomitant immunomodulatory therapy (IMT) were analyzed.

Among 190 AAA-positive patients, 18 (9.47%) demonstrated antibody resolution over a median follow-up of 6.5 months. These patients had lower median AAA levels (39.55 ng/mL vs. 92.35 ng/mL, *p*=0.020) and higher adalimumab levels (6.25 μg/mL vs. 1.55 μg/mL, *p*=0.018) than controls. AAA resolution was negatively correlated with AAA levels (*p*=0.018) and positively correlated with adalimumab levels (*p*=0.016).

**Conclusions:**

Therapeutic monitoring of AAA and drug levels may help guide personalized therapeutic strategies and support continued treatment in selected patients.

## Key points

I. Therapeutic drug monitoring enables individualized management by identifying patients with low AAA and adequate drug levels who may safely continue adalimumab.

II. Detection of AAA should not lead to automatic discontinuation; Therapeutic drug monitoring can guide personalized decisions to maintain effective treatment.

## Introduction

Biological agents targeting tumor necrosis factor-alpha (TNF-α), notably adalimumab, represent some of the most effective steroid-sparing therapies available. The US Food and Drug Administration (FDA) has approved adalimumab for systemic autoimmune conditions, including inflammatory arthritis and inflammatory bowel disease (IBD), as well as for ocular inflammatory disease (OID) [1–6]. 

Adalimumab (Humira^®^, AbbVie Inc., North Chicago, IL, USA) is a fully humanized monoclonal antibody binding to both monomeric and trimeric TNF-α. It inhibits the abnormal immune response by targeting essential inflammatory mediators to halt the progression of the disease [1]. 

Adalimumab, like other antibody-based biologics, is prone to the development of neutralizing anti-drug antibodies (ADAb) by the host [7]. ADAb may develop through two main immune pathways: a T-cell-dependent process, where antigen-presenting cells stimulate CD4 + T cells and trigger high-affinity IgG production, and a T-cell-independent pathway, where direct B-cell receptor engagement leads to a faster but shorter-lived IgM response [8]. These immune mechanisms, influenced by patient-specific and treatment-related factors, may help explain the variability in AAA persistence and resolution observed in clinical practice [8]. Thus, therapeutic drug monitoring (TDM) is essential, requiring the measurement of both drug levels and ADAb [9, 10]. The American Gastroenterological Association (AGA) recommends an optimal adalimumab trough concentration of ≥ 7.5 µg/ml.^10^ Previous research has identified risk factors for immunogenicity and the clinical implications of AAA [11–13]. 

Despite extensive research that has been conducted on adalimumab and AAA, there is limited data regarding the best management strategy. If an AAA were to be identified during the course of treatment, the question to whether continue adalimumab or switch to alternative treatment modality, particularly in patients who were initially responding well to adalimumab can produce a treatment limbo [14, 15]. 

The index study analyzed the demographic data and characteristics of patients who continued adalimumab therapy with increased dose or frequency despite developing AAA with subsequent resolution of AAA during follow-up, aiming to explore the association with these transitory antibodies.

## Methods

### Study design and patient recruitment

Medical records from the STAnford Research Repository (STARR), spanning from June 2006 to May 2024, were reviewed to identify patients who received adalimumab for various autoimmune conditions and developed AAA. The research was conducted in accordance with the Declaration of Helsinki, the United States Code of Federal Regulations Title 21, and the Harmonized Tripartite Guidelines for Good Clinical Practice (1996). As the study involved a retrospective review of patient records, an informed consent waiver was obtained for enrolled patients.

Patients were included in the study only if they initially tested positive for AAA and later tested negative and were considered as the Case group. Cases were then matched with a Control group consisting of patients who remained positive for AAA. Matching was conducted based on age, sex, and systemic disease. Patients with insufficient data, those lost to follow-up, or those whose antibody and drug levels were measured within three days of adalimumab administration were excluded.

The demographic data and characteristics of the patients who showed transitory AAA were collected and analyzed including their clinical diagnosis, adalimumab dosage and frequency, trough adalimumab level, serum AAA level, and any concomitant immunomodulatory therapy (IMT).

### Measurement of serum adalimumab trough and AAA levels

For patients on adalimumab therapy, routine therapeutic drug monitoring (TDM) is conducted by measuring serum trough levels to determine if they are therapeutic or subtherapeutic. If the trough level is 8 µg/ml or less, testing for anti-adalimumab antibodies (AAA) is performed. Both adalimumab trough levels and AAA are measured using an Enzyme-Linked Immunosorbent Assay (ELISA) at Quest Diagnostics™. AAA is considered positive if titers are ≥ 10 ng/ml in a single measurement [16].

The first detectable serum AAA level in both case and control groups were recorded simultaneously with the trough adalimumab level. Serum levels of both adalimumab trough and AAA were reassessed during three consecutive visits, approximately three months apart.

### Statistical analysis

The collected data were organized, tabulated, and statistically analyzed using the IBM^®^ SPSS statistical software, version 21 (Statistical Package for Social Studies) created by IBM, Illinois, Chicago, USA. In this study, the qualitative data were described using number and percentage. We utilized the one-sample Shapiro - Wilk test to check the normality of data and some of data were parametric and others were non-parametric. Quantitative data were presented as median and interquartile range (IQR). P value of ≤ 0.05 was used as a cut off value for significance of results.

The Chi-square test was used to compare categorical variables of different groups with Fisher’s Exact or Monte Carlo correction for Chi-square when more than 20% of the cells have expected count less than 5. Moreover, the Mann Whitney test was applied to compare abnormally distributed quantitative variables between two studied groups.

For studying the direction and strength of the relationship between two variables (continuous nonparametric and categorical), Spearman correlation coefficient (r_s_) was employed.

## Results

### Study population and baseline characteristics

Seven hundred and ninety (790) patients were identified receiving adalimumab for IBD, OID, inflammatory arthritis, and other autoimmune diseases. Upon reviewing their charts, we found that AAA was detectable in 190 patients. Of these, 18 (9.47%) showed a resolution of AAA during median follow up of 6.5 months and were categorized as the case group. We included additional 18 patients with persistent AAA as a control group, matched for age, sex and systemic disease.

The mean age of the cases was 22.50 ± 16.69 years and 11 were female (61.1%). Crohn’s disease was the most prevalent underlying disease, affecting 10 patients (55.6%) followed by ulcerative colitis and mixed autoimmune conditions, each affecting 3 (16.7%) respectively. In total 3 patients (16.7%) were diagnosed with Ocular Inflammatory Disease (OID); one had juvenile idiopathic arthritis-associated uveitis, another presented with nodular episcleritis in conjunction with Crohn’s disease and the third had multifocal choroiditis and panuveitis without any identified systemic disease.

Patients in both groups were treated with adalimumab as monotherapy or in combination with other IMT such as methotrexate or (MTX), azathioprine (AZA).

Out of the 18 cases, 11 (61.1%) were receiving IMT along with adalimumab before AAA development-10 on MTX and 1 on AZA, while only 8 of the control patients (44.44%) were on IMT, seven were on MTX and 1 on AZA, with no significant difference between the two groups (*p* = 0.589). A detailed description of the demographics, clinical diagnoses, and IMT in the two studied groups are provided in Table 1.

**Table 1:** Comparison between cases and match groups regarding demographic data, diagnosis and Immunomodulatory therapy (IMT)

Variable	Cases *n* = 18	Match *n* = 18	Test of sig.	*P*
Age / years				
Range	10–69	11–56	U	0.131
Mean ± SD	22.50 ± 16.69	23.94 ± 11.85		
Median (IQR)	17.00 (12 −24.75)	21 (17–23)	114.50	
Sex / n (%)				
Male	7 (38.9)	7 (38.9)	χ2	0.182
Female	11(61.1)	11(61.1)	1.778	
Race / n (%)				
Asian population	4(22.2)	1(5.6)		
Other population	5(27.8)	7 (38.9)	MC	
Unknown population	2(11.1)	2 (11.1)		0.472
White population	7(38.9)	6 (33.3)		
Black population	0(0)	2 (11.1)		
Diagnosis / n (%)				
Crohn’s disease	10(55.6)	12(66.7)		
Ulcerative colitis	3(16.7)	3(16.7)		
JIA	1(5.6)	1(5.6)	MC	
Mixed	3(16.7)	2(11.2)		
Multifocal choroiditis and panuveitis without systemic disease	1(5.6)	0(0)		

*U *Mann-Whitney test χ2: Chi square test *MC *Monte-Carlo test *P P* value

Of note, only one patient from the case group was on another TNF-alpha inhibitor (etanercept) before starting adalimumab while 4 patients from the control group were receiving other TNF inhibitor (2 etanercept, 2 infliximab) before switching to adalimumab. The majority of patients 13 (72.22%) from each group were on 40 mg adalimumab every 2 weeks before developing AAA while the remaining were either on 20 mg every 2 weeks (2 cases, 2 controls) or 40 mg weekly (3 cases, 3 controls).

### Serum trough adalimumab and AAA levels

The American Gastroenterological Association (AGA) recommends a target trough concentration of ≥ 7.5 µg/mL for adalimumab in patients with active IBD on maintenance therapy [11]. In our study, most patients in both the case and control groups exhibited subtherapeutic trough levels, with median values of 6.25 and 1.55 µg/ml, respectively. Additionally, both groups had detectable serum levels of AAA (≥ 10 ng/ml), with median values of 39.55 in case and 92.35 ng/ml in control group (*P* = 0.02) s shown in Table 2.

**Table 2:** Comparison between cases and match groups regarding duration of adalimumab therapy from start till first AAA positivity, and from first AAA positivity till negativity, adalimumab blood level, AAA levels at the first AAA positivity and IMT intake during period of conversion

Variable	Cases *n* = 18	Match *n* = 18	Test of significance	*P*
	Range Median (IQR) Mean ± SD			
Duration of adalimumab therapy from start until first AAA positivity (months)	2 – 53	2– 106	U	
	7(3 – 23.25)	14(4.75 – 45.75)	124.00	0.228
	14.44± 16.94	28.11± 31.66		
Adalimumab blood level (μg /ml) at the first AAA positivity	<0.8 – 20	<0.8 – 21.70	U	
	6.25(3.17 – 8.60)	1.55(0.70 – 6.20	87.50	0.018
	6.62± 4.80	4.01± 5.40		
Levels of AAA at the first-time of positivity (ng/ml)	11 −166	10 - >501	U	
	39.55(16.80 – 79.98)	92.35(47.75 – 113.20)	88.50	0.020
	53.25± 43.24	126.58± 142.11		
Duration of adalimumab therapy from first AAA positivity until negativity (months)	1 - 29			
	6.50(3 – 12.25)			
	9.44± 8.10			
IMT intake during period of conversion				
Yes	9	5	**χ2**	0.392
No	2	3	1.872	

*U *Mann-Whitney test *P P* value

Cases developed AAA in a shorter duration than controls (median: 7 months vs. 14 months), although this difference was not statistically significant. (*p* = 0.228) Table 2.

### Disease activities at time of adalimumab antibodies detection

Among cases, 77.8% of patients were in a stable disease state, compared to 55.6% in the control group (*p* = 0.289). Conversely, disease activity was observed in 22.2% of cases and 44.4% of controls.

### Action taken after detection of AAA

**Fig. 1:**
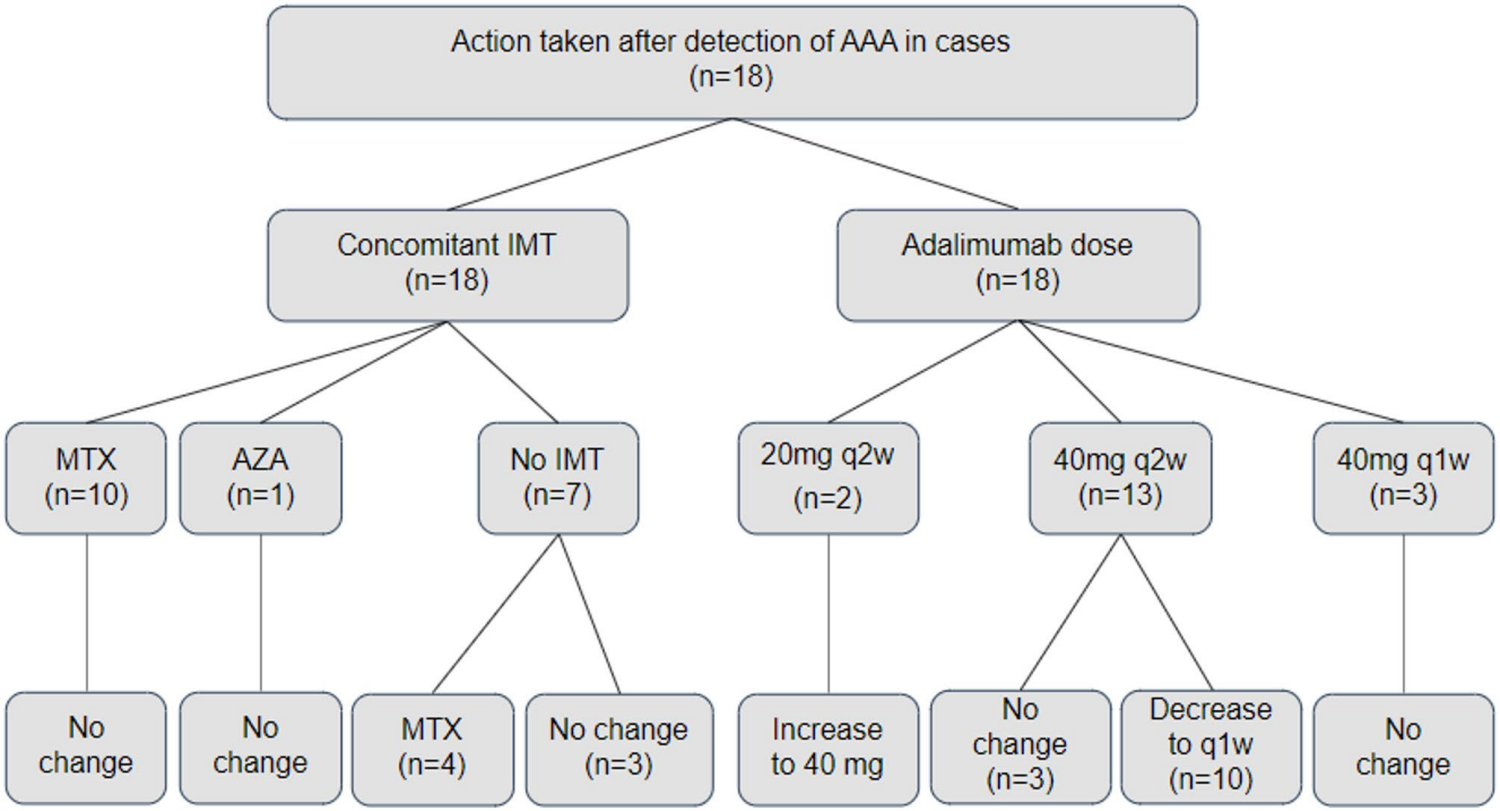
Flow chart showing adalimumab and immunomodulatory therapies in cases, and the actions that had been taken after AAA development. AAA: Anti-adalimumab antibodies; IMT: Immune Modulator Therapy; MTX: Methotrexate; AZA: Azathioprine

Among the cases, the adalimumab dose was increased to 40 mg every 2 weeks in the 2 cases who were on 20 mg adalimumab every 2 weeks. Furthermore, the dosing interval was reduced to weekly instead of biweekly in 10 of the 13 cases who were on 40 mg adalimumab every 2 weeks. Given the stability of the disease in the remaining 3 cases, they continued on 40 mg adalimumab every 2 weeks with no modification in the dose or timing of adalimumab administration. Regarding IMT, MTX was added in 4 cases along with adalimumab as shown in Fig. 1.

In the control group, high level of AAA persisted in 2 consecutive tests done approximately 3 months apart, despite increasing the dose of adalimumab, reducing the dosing interval and/or adding IMT. Consequently, 9 patients switched to an alternative biologic because their disease was uncontrolled, while the remaining 9 continued treatment with adalimumab, as their disease was controlled despite presence of AAA.

### Resolution of AAA in cases and comparison between the two study groups

The median duration for AAA to negative or below the level of detection was 6.50 (range: 1–29) months as shown in Table 2. After the absence of AAA, 15 cases continued adalimumab therapy with optimum disease control and AAA remained negative in 2 consecutive tests conducted about 3 months apart. The remaining 3 cases switched to another biologic (vedolizumab, infliximab, ustekinumab) due to active disease status despite negative AAA.

Notably, the median serum level of first detectable AAA was significantly lower in cases (39.55 ng/mL) than in controls (92.35 ng/mL) *(p* = 0.020) along with significantly higher median serum adalimumab trough level (*p* = 0.018) as shown in Table 2.

**Fig. 2:**
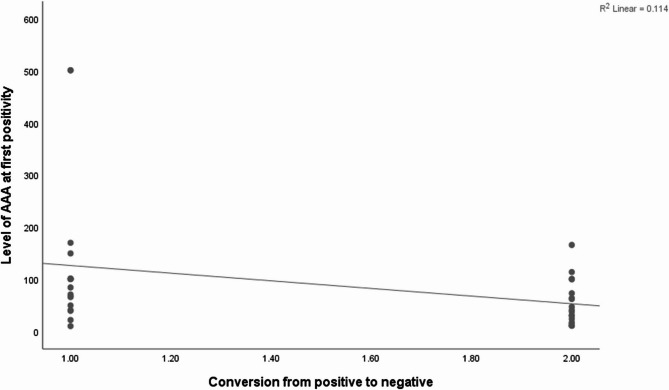
Scatterplot graph shows significant negative correlation between level of AAA at first positivity and conversion of AAA (*p* = 0.018, r_s_=−0.394)

**Fig. 3:**
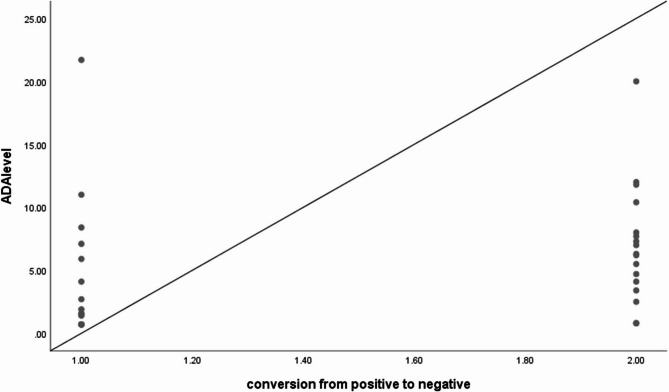
The scatterplot graph shows a significant positive correlation between ADA level and conversion of AAA (*p* = 0.016, r_s_=0.400)

Furthermore, a significant negative correlation was noticed between AAA level and resolution of AAA (*p* = 0.018, *rs*=−0.394) as demonstrated in Fig. 2. There was also a significant positive correlation between serum adalimumab level at time of first detectable AAA and the disappearance of AAA (*p* = 0.016, *rs* = 0.400) as demonstrated in Fig. 3.

A negative correlation was detected between the resolution of AAA and duration of adalimumab therapy till detection of AAA, but it was statistically non-significant (*p* = 0.235, *rs*=−0.203).

## Discussions

With the increasing use of adalimumab in treating autoimmune disorders either systemic such as IBS, rheumatologic disorders or OID, studies have been conducted to determine the clinical response to adalimumab and its therapeutic level [17]. Despite providing excellent control of inflammation, it has been observed in many of these studies that the response to adalimumab among individuals can vary, with some even experiencing flares despite an initially favorable outcome. It is now understood that one reason for this loss of response is the development of neutralizing AAA [16, 18]. These anti-adalimumab antibodies (AAA) can inhibit the drug’s efficacy or reduce its bioavailability [19]. The development of anti-drug antibodies (ADA) is influenced by a complex interplay of drug-related, patient-specific, and regimen-related factors [20]. Host-related factors play a significant role in the variability of AAA development and resolution observed among patients. Specific HLA class II haplotypes have been linked to either a higher or lower risk of developing AAA in those receiving adalimumab, underscoring the contribution of genetic predisposition to immunogenicity [21]. Additionally, lower baseline levels of TNF-α have been associated with a higher likelihood of ADA formation, likely due to increased availability of unbound adalimumab acting as a target for immune recognition. In our cohort, we observed considerable heterogeneity in AAA titers and clinical trajectories, which may reflect these underlying immunogenetic and disease-specific differences. Regimen-related factors appear to play a critical role in the development and resolution of AAA. Sustained or high dosing of adalimumab may promote immune tolerance, while subtherapeutic exposure or prolonged dosing intervals can increase immunogenicity risk [22, 23]. In our cohort, several patients with initial AAA positivity experienced antibody clearance and maintained clinical stability, particularly following interventions such as dose escalation or the addition of immunomodulatory agents. These findings reinforce the importance of a tailored therapeutic strategy that considers individual drug exposure, immune response, and treatment history. The behavior of anti-adalimumab antibodies (AAAs) may be influenced by immune complex size, antibody isotype, and disease-specific clearance mechanisms [24]. Smaller immune complexes tend to persist and trigger further immune activation, while larger ones are cleared more rapidly [24]. Additionally, persistent AAAs are often IgG1 or IgG4, with IgG4 linked to prolonged antigen exposure. These factors, along with differences in immune responses across diseases like Crohn’s and ocular inflammation, may partly explain the variability in AAA persistence and treatment outcomes [24]. 

However, there is no consensus on how to treat patients with immune-mediated subtherapeutic trough levels and positive AAA, where ADAb-drug complex accelerates drug clearance/ neutralization and directly prevents the drug from binding TNF. One of the common approaches adopted is to shift to another biologic despite favorable response to adalimumab at the start of therapy [14, 19]. 

We hereby studied 36 patients treated with adalimumab for various autoimmune conditions with consequent subtherapeutic trough level and AAA development. Of these, 18 had transient AAA (case group) and 18 (control group) had persistent AAA. We compared both groups to better understand which patients might benefit from maintaining adalimumab therapy despite positive AAA and which should switch to another biologic therapy.

About half of patients in both groups received concomitant treatment with IMT in the form of MTX or AZA with no significant difference between the two groups (*p* = 0.589). This finding suggests that the protective effect of IMT on adalimumab immunogenicity is not universal and may or may not prevent the development of AAA. Similarly, other studies reported no significant difference in AAA formation between patients with and without concomitant IMT [9, 12]. In contrast, previous literature demonstrated that IMT, particularly MTX, can reduce the incidence of AAA formation [11, 13, 25]. Although inconclusive and requiring further exploration, we hypothesize from our observation that IMT may help in delaying but *not* completely preventing the formation of AAA. Moreover, a history of prior TNF-alpha inhibitor use could potentially be a risk factor for AAA development given that 4 controls and 1 case had previously been treated with another TNF-alpha inhibitor, which may contribute to increased immunogenicity [26]. 

AAA were identified in both groups over a broad treatment duration range (2–53 months in cases and 2–106 months in controls). Such finding aligns with McKay KM et al.‘s findings, which reported treatment durations ranging from 5 to 76 months [19]. However, AAA development occurred earlier in cases compared to controls but was not statistically significant (*p* = 0.228). This observation suggests that the presence of AAA cannot be predicted by the treatment duration.

Our findings suggest that nearly all cases and many controls (9 patients) did not experience flare up and had good control of the inflammation despite the presence of AAA which justified the continuation of adalimumab therapy. This outcome suggests that AAA development does not mean therapeutic failure which can be explained by the dynamic process of ADAb formation where the immune system may produce different subclasses of ADAb or alter their affinity for the drug. As a result, AAA did not fully neutralize *all* the available adalimumab and/or were directed against non-relevant epitopes.

In contrast, Bartelds et al. noticed that AAA was related to diminished drug bioavailability and decreased clinical efficacy [27]. The discrepancy in findings could be attributed to the small patient sample, the variety of immunosuppressive drugs used, and the inclusion of different types of underlying diseases [27]. 

Previous studies have indicated that AAA development was associated with a reduced adalimumab level [11, 14, 18, 27]. Our results, however, suggest that cases demonstrated a significantly higher median trough adalimumab level, and a much lower median AAA level compared to controls with *P* value 0.018 and 0.020, respectively. In a consistent manner, Cordero-Coma M, et al. reported that patients with persistent AAA had much higher AAA titers and lower trough adalimumab levels compared to those with transitory AAA [12]. Consistent with these findings, we observed a significant negative correlation between AAA levels and AAA resolution, as well as a significant positive correlation between trough adalimumab levels and AAA disappearance, with P-values of 0.018 and 0.016, respectively.

Our findings indicate that proactive TDM might be useful in optimizing treatment and potentially preventing flares and loss of response in patients who have already shown some therapeutic response. Similarly, previous studies advocate routine monitoring of drug levels and ADAb as a clinical and cost-effective approach to personalize anti-TNF therap [28, 29]. This monitoring enables the early detection of low AAA levels which may be countered by increasing drug dose, shortening of dosing interval and/or the addition of IMT rather than discontinuation of adalimumab. Our findings are also consistent with the study by Pichi et al., which demonstrated that therapeutic drug monitoring, dose escalation, and the use of concomitant immunomodulatory agents may reverse immunogenic loss of response in noninfectious uveitis [30]. Their work supports the idea that AAA behavior can be modified through individualized strategies, in line with our observed association between lower AAA titers, higher adalimumab levels, and subsequent antibody resolution. Although tapering of adalimumab was not observed in our retrospective cohort, this remains an important consideration for long-term management. Several patients became antibody-negative and remained clinically stable, raising the question of whether dose reduction could be a viable strategy in selected cases. In the *PREDICTRA trial*, 64% of patients with rheumatoid arthritis remained flare-free after adalimumab dose tapering, while 35% experienced a flare, and only about half of those regained disease control with rescue therapy [31]. *The STRASS* study, which used a stepwise tapering approach, reported similar results, with approximately one-third of patients relapsing during de-escalation [32]. These findings highlight that while tapering may be feasible for some patients in sustained remission with high drug levels, predicting who will respond well remains challenging. Our data suggest that patients with low AAA levels and high trough concentrations may warrant further study as potential candidates for safe and effective tapering strategies.

TDM is crucial to distinguish between treatment failure caused by pharmacokinetic factors, where the drug is undetectable, and pharmacodynamic factors, where the drug is present but ineffective. An optimal therapeutic adalimumab trough level indicates TNF-independent disease and switching to a non-TNF blocker should be considered. On the other hand, subtherapeutic trough level may be detected with or without the presence of AAA. Managing patients who had subtherapeutic trough level and negative AAA due to non-immune pharmacokinetic issues involves ensuring adherence to treatment, adjusting the dose, or shortening the dosing interval to reduce drug clearance [14, 16, 17]. 

### Strengths and limitations

We realize that our study is considered exploratory and has certain limitations. Firstly, the small sample size despite reviewing medical records of 790 patients receiving adalimumab for various autoimmune conditions. Among these, 190 tested positive for AAA, and only 18 later became negative. Large-scale, multicenter prospective studies are needed to validate and confirm our findings. Secondly, the heterogeneity of the disease types included in the study further limits the ability to generalize the results. Nevertheless, we believe the data is relevant and represents a step forward. The findings of this study contribute to the limited existing literature on AAA and demonstrate that adalimumab therapy can still be successfully continued despite the presence of AAA with subsequent resolution of these antibodies.

Further studies are required to explore the correlation between the trough level of adalimumab, the presence of AAA, and their clinical impact on disease status, particularly in OID.

## Conclusion

Our study emphasizes the importance of TDM during adalimumab therapy. It highlights potential strategies to overcome AAA and salvage adalimumab therapy rather than switching to alternative treatment modality, particularly in patients who had higher trough adalimumab level and/or lower median AAA as these antibodies can be transitory. The presence of AAA should not be considered the end point of the use of adalimumab, especially in cases where the disease is controlled despite its presence. Our index study shows that the reversal of presence of AAA is possible and paves a way for future studies to understand the mechanism even better.

## Data Availability

No datasets were generated or analysed during the current study.

## Abbreviations

AAA: Anti-adalimumab Antibodies
ADA: Adalimumab
ADAb: Anti-drug Antibodies
AGA: American Gastroenterological Association
AZA: Azathioprine
ELISA: Enzyme-Linked Immunosorbent Assay
FDA: Food and Drug Administration
IBD: Inflammatory Bowel Disease
IBM SPSS: IBM Statistical Package for the Social Sciences
IMT: Immunomodulatory Therapy
IQR: Interquartile Range
JIA: Juvenile Idiopathic Arthritis
MTX: Methotrexate
NIU: Non-Infectious Uveitis
OID: Ocular Inflammatory Disease
STARR: Stanford Research Repository
TDM: Therapeutic Drug Monitoring
TNF-α: Tumor Necrosis Factor-alpha

## References

[1] Breedveld FC, Weisman MH, Kavanaugh AF et al (2006) The PREMIER study: a multicenter, randomized, double‐blind clinical trial of combination therapy with adalimumab plus methotrexate versus methotrexate alone or adalimumab alone in patients with early, aggressive rheumatoid arthritis who had not had previous methotrexate treatment. Arthritis Rheum 54(1):26–37. 10.1002/art.21519 16385520 10.1002/art.21519

[2] Colombel JF, Sandborn WJ, Ghosh S et al (2014) Four-year maintenance treatment with adalimumab in patients with moderately to severely active ulcerative colitis: data from ULTRA 1, 2, and 3. Am J Gastroenterol 109(11):1771–1780. 10.1038/ajg.2014.242 25155227 10.1038/ajg.2014.242 PMC4223868

[3] Dunn BD, Widnall E, Reed N et al (2019) Evaluating augmented depression therapy (ADepT): study protocol for a pilot randomised controlled trial. Pilot Feasibility Stud 5:63. 10.1186/s40814-019-0438-1 31061718 10.1186/s40814-019-0438-1 PMC6486988

[4] Schreiber S, Sandborn WJ (2006) CLASSIC-I study the efficacy of adalimumab. Gastroenterology 130(6):1929–1930. 10.1053/j.gastro.2006.03.050 16697761 10.1053/j.gastro.2006.03.050

[5] Jaffe GJ, Dick AD, Brézin AP et al (2016) Adalimumab in patients with active noninfectious uveitis. N Engl J Med 375(10):932–943. 10.1056/NEJMoa1509852 27602665 10.1056/NEJMoa1509852

[6] Nguyen QD, Merrill PT, Jaffe GJ et al (2016) Adalimumab for prevention of uveitic flare in patients with inactive non-infectious uveitis controlled by corticosteroids (VISUAL II): a multicentre, double-masked, randomised, placebo-controlled phase 3 trial. Lancet 388(10050):1183–1192. 10.1016/S0140-6736(16)31339-3 27542302 10.1016/S0140-6736(16)31339-3

[7] Thomas SS, Borazan N, Barroso N et al (2015) Comparative immunogenicity of TNF inhibitors: impact on clinical efficacy and tolerability in the management of autoimmune diseases. A systematic review and meta-analysis. BioDrugs 29(4):241–258. 10.1007/s40259-015-0134-5 26280210 10.1007/s40259-015-0134-5

[8] Howard EL, Goens MM, Susta L, Patel A, Wootton SK (2025) Anti-drug antibody response to therapeutic antibodies and potential mitigation strategies. Biomedicines 13(2):299. 10.3390/biomedicines13020299 40002712 10.3390/biomedicines13020299 PMC11853408

[9] Kato M, Sugimoto K, Ikeya K et al (2021) Therapeutic monitoring of adalimumab at non-trough levels in patients with inflammatory bowel disease. PLoS One 16(7):e0254548. 10.1371/journal.pone.0254548 34242369 10.1371/journal.pone.0254548 PMC8270420

[10] Papamichael K, Vande Casteele N, Ferrante M, Gils A, Cheifetz AS (2017) Therapeutic drug monitoring during induction of anti-tumor necrosis factor therapy in inflammatory bowel disease: defining a therapeutic drug window. Inflamm Bowel Dis 23(9):1510–1515. 10.1097/MIB.0000000000001231 28816757 10.1097/MIB.0000000000001231

[11] Bellur S, McHarg M, Kongwattananon W, Vitale S, Sen HN, Kodati S (2023) Antidrug antibodies to tumor necrosis factor α inhibitors in patients with noninfectious uveitis. JAMA Ophthalmol 141(2):150–156. 10.1001/jamaophthalmol.2022.5584 36547953 10.1001/jamaophthalmol.2022.5584 PMC9936342

[12] Cordero-Coma M, Calleja-Antolín S, Garzo-García I et al (2016) Adalimumab for treatment of noninfectious uveitis: immunogenicity and clinical relevance of measuring serum drug levels and antidrug antibodies. Ophthalmology 123(12):2618–2625. 10.1016/j.ophtha.2016.08.025 27692527 10.1016/j.ophtha.2016.08.025

[13] Bromeo AJ, Karaca I, Ghoraba HH et al (2024) Risk factors for development of anti-adalimumab antibodies in non-infectious uveitis. Heliyon 10(9):e29313. 10.1016/j.heliyon.2024.e29313 38694084 10.1016/j.heliyon.2024.e29313 PMC11061690

[14] Ding NS, Hart A, De Cruz P (2016) Systematic review: predicting and optimising response to anti-TNF therapy in crohn’s disease - algorithm for practical management. Aliment Pharmacol Ther 43(1):30–51. 10.1111/apt.13445 26515897 10.1111/apt.13445

[15] Kothari MM, Nguyen DL, Parekh NK (2017) Strategies for overcoming anti-tumor necrosis factor drug antibodies in inflammatory bowel disease: case series and review of literature. World J Gastrointest Pharmacol Ther 8(3):155–161. 10.4292/wjgpt.v8.i3.155 28828193 10.4292/wjgpt.v8.i3.155 PMC5547373

[16] Sejournet L, Kerever S, Mathis T, Kodjikian L, Jamilloux Y, Seve P (2022) Therapeutic drug monitoring guides the management of patients with chronic non-infectious uveitis treated with adalimumab: a retrospective study. Br J Ophthalmol 106(10):1380–1386. 10.1136/bjophthalmol-2021-319072 33875451 10.1136/bjophthalmol-2021-319072

[17] Cludts I, Spinelli FR, Morello F, Hockley J, Valesini G, Wadhwa M (2017) Anti-therapeutic antibodies and their clinical impact in patients treated with the TNF antagonist adalimumab. Cytokine 96:16–23. 10.1016/j.cyto.2017.02.015 28279855 10.1016/j.cyto.2017.02.015 PMC5484178

[18] Jyssum I, Gehin JE, Sexton J et al (2024) Adalimumab serum levels and anti-drug antibodies: associations to treatment response and drug survival in inflammatory joint diseases. Rheumatology (Oxford) 63(6):1746–1755. 10.1093/rheumatology/kead525 37773994 10.1093/rheumatology/kead525 PMC11147536

[19] McKay KM, Apostolopoulos N, Chou B, Leveque TK, Van Gelder RN (2022) Anti-adalimumab antibodies in patients with non-infectious ocular inflammatory disease: a case series. Ocul Immunol Inflamm 30(7–8):1721–1725. 10.1080/09273948.2021.1936565 34270383 10.1080/09273948.2021.1936565

[20] Nabhan M, Pallardy M, Turbica I (2020) Immunogenicity of bioproducts: cellular models to evaluate the impact of therapeutic antibody aggregates. Front Immunol 11:725. https://www.frontiersin.org/journals/immunology/articles/10.3389/fimmu.2020.00725/full [Google Scholar] [CrossRef] [PubMed] - Google Search. Accessed June 24, 2025 32431697 10.3389/fimmu.2020.00725 PMC7214678

[21] Mosch R, Guchelaar HJ (2022) Immunogenicity of monoclonal antibodies and the potential use of HLA haplotypes to predict vulnerable patients. Front Immunol 13:885672. 10.3389/fimmu.2022.885672 35784343 10.3389/fimmu.2022.885672 PMC9249215

[22] Vultaggio A, Perlato M, Nencini F, Vivarelli E, Maggi E, Matucci A (2021) How to prevent and mitigate hypersensitivity reactions to biologicals induced by anti-drug antibodies?? Front Immunol 12:765747. 10.3389/fimmu.2021.765747 34790200 10.3389/fimmu.2021.765747 PMC8591239

[23] Shepard HM, Phillips GL, Thanos D, Feldmann C (2017) Developments in therapy with monoclonal antibodies and related proteins. Clin Med (Lond) 17(3):220–232. 10.7861/clinmedicine.17-3-220 28572223 10.7861/clinmedicine.17-3-220 PMC6297577

[24] Krishna M, Nadler SG. Immunogenicity to Biotherapeutics – The role of Anti-drug immune complexes. Front Immunol. 2016;7. 10.3389/fimmu.2016.00021 10.3389/fimmu.2016.00021 PMC4735944 26870037

[25] Krieckaert CL, Nurmohamed MT, Wolbink GJ (2012) Methotrexate reduces immunogenicity in adalimumab treated rheumatoid arthritis patients in a dose dependent manner. Ann Rheum Dis 71(11):1914–1915. 10.1136/annrheumdis-2012-201544 22586169 10.1136/annrheumdis-2012-201544

[26] Kalden JR, Schulze-Koops H (2017) Immunogenicity and loss of response to TNF inhibitors: implications for rheumatoid arthritis treatment. Nat Rev Rheumatol 13(12):707–718. 10.1038/nrrheum.2017.187 29158574 10.1038/nrrheum.2017.187

[27] Bartelds GM, Wijbrandts CA, Nurmohamed MT et al (2007) Clinical response to adalimumab: relationship to anti-adalimumab antibodies and serum adalimumab concentrations in rheumatoid arthritis. Ann Rheum Dis 66(7):921–926. 10.1136/ard.2006.065615 17301106 10.1136/ard.2006.065615 PMC1955110

[28] Laine J, Jokiranta TS, Eklund KK, Väkeväinen M, Puolakka K (2016) Cost-effectiveness of routine measuring of serum drug concentrations and anti-drug antibodies in treatment of rheumatoid arthritis patients with TNF-α blockers. Biologics 10:67–73. 10.2147/BTT.S96982 27099470 10.2147/BTT.S96982 PMC4824281

[29] Negoescu DM, Enns EA, Swanhorst B et al (2020) Proactive vs reactive therapeutic drug monitoring of Infliximab in Crohn’s disease: a cost-effectiveness analysis in a simulated cohort. Inflamm Bowel Dis 26(1):103–111. 10.1093/ibd/izz113 31184366 10.1093/ibd/izz113 PMC6905301

[30] Pichi F, Smith SD, AlAli SH, Neri P (2024) Adalimumab drug monitoring and treatment adjustment to drug antibodies in noninfectious uveitis. Am J Ophthalmol 268:306–311. 10.1016/j.ajo.2024.09.008 39271091 10.1016/j.ajo.2024.09.008

[31] Emery P, Burmester GR, Naredo E et al (2020) Adalimumab dose tapering in patients with rheumatoid arthritis who are in long-standing clinical remission: results of the phase IV predictra study. Ann Rheum Dis 79(8):1023–1030. 10.1136/annrheumdis-2020-217246 32404343 10.1136/annrheumdis-2020-217246 PMC7392484

[32] Marotte H, Rinaudo-Gaujous M, Petiet C, Fautrel B, Paul S (2020) Tapering without relapse in rheumatoid arthritis patients with high TNF blocker concentrations: data from STRASS study. Ann Rheum Dis 79(7):e81. 10.1136/annrheumdis-2019-215546 31048287 10.1136/annrheumdis-2019-215546

